# Supplementary material for: Identification of photoperiod-induced specific miRNAs in the adrenal glands of Sunite sheep (Ovis aries)
Source: Front Vet Sci. 2022 Jul 22;9:888207. doi: 10.3389/fvets.2022.888207 (PMC9354845; doi:10.3389/fvets.2022.888207)
Supplement: Supplementary file 1 [file Data_Sheet_1.ZIP › Supplementary file/miRDeep2 file.zip!/miRDeep2 file/Know_Structure/LP42A2/oar-mir-29a.pdf]

miRBase precursor : oar-mir-29a
Total read count : 145791
oar-miR-29a read count : 145756
remaining reads : 35

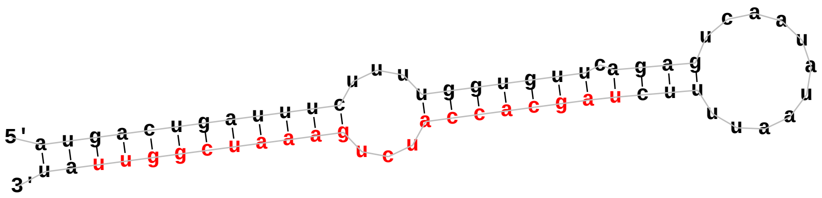

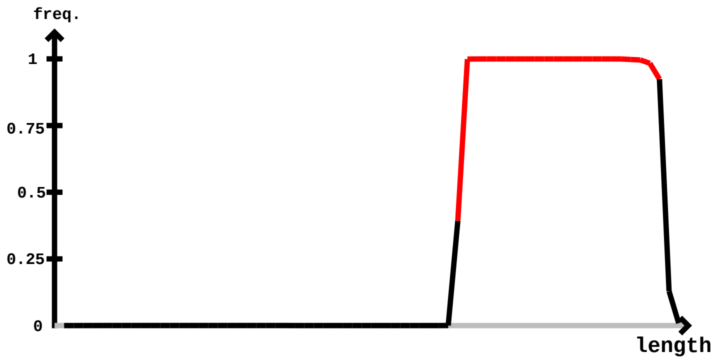


```
                                                                      oar-miR-29a
5'- augacugauuucuuuugguguucagagucaauauaauuuucuagcaccaucugaaaucgguuau   -3'    exp
    ((((((((((((...(((((((.((((...........)))))))))))...))))))))))))    reads      mm         sample
    .ugacugauuucuuuugguguuc.........................................    16         0          seq
    ...acugauuucuuuugguguu..........................................    1          0          seq
    ...acugauuucuuCgguguuc..........................................    1          1          seq
    ...acugauuucuuuugguguuc.........................................    15         0          seq
    .....ugauuucuuuugguguuU.........................................    2          1          seq
    .......................................ucuagcaccaucugaaauc......    1          0          seq
    .......................................ucuagcaccaucugaaaucgg....    5          0          seq
    .......................................ucuagcaccaucugaaaucggu...    20         0          seq
    .......................................ucuagcaccaucugaaaucgguu..    4          0          seq
    ........................................cuagcaccaucugaaaA.......    3          1          seq
    ........................................Auagcaccaucugaaau.......    1          1          seq
    ........................................cuCgcaccaucugaaau.......    1          1          seq
    ........................................cuagcaccaucugaaau.......    8          0          seq
    ........................................cuagcaccaucugaaauc......    55         0          seq
    ........................................cuagcaccaucugaaauA......    4          1          seq
    ........................................cuagcaccaucugaaaucg.....    53         0          seq
    ........................................cuagcaccaucugaaaucgg....    286        0          seq
    ........................................cuagcaccauUugaaaucggu...    26         1          seq
    ........................................cuagcaccaucugaaaucggu...    2397       0          seq
    ........................................cuagcaccauUugaaaucgguu..    235        1          seq
    ........................................cuagcaccaucugaaaucgguu..    53006      0          seq
    ........................................cuagcaccaucugaaaucgguua.    1075       0          seq
    ........................................cuagcaccauUugaaaucgguua.    5          1          seq
    ........................................cuagcaccaucugaaaucgguuau    68         0          seq
    .........................................uagcaccaucugaaauU......    24         1          seq
    .........................................uagcaccaucugaaauc......    195        0          seq
    .........................................uGgcaccaucugaaauc......    1          1          seq
    .........................................uagcacUaucugaaauc......    1          1          seq
    .........................................uagcaccaucugaaauA......    9          1          seq
    .........................................uagcaccaucugaaGuc......    1          1          seq
    .........................................uagGaccaucugaaauc......    1          1          seq
    .........................................uagcaccauUugaaaucg.....    17         1          seq
    .........................................uagcaccaucugaaaucg.....    180        0          seq
    .........................................uagcaccaucugaaaucgg....    1392       0          seq
    .........................................uagcaccauUugaaaucgg....    126        1          seq
```

oar-miR-29a

```
augacugauuucuuuugguguucagagucaauauaauuuucuagcaccaucugaaaucgguuau
.........................................uagcaccauUugaaaucggu...     756       1          seq
.........................................uagcaccaucugaaaucggu...     5468      0          seq
.........................................uagcaccaucugaaaucgguu..     59431     0          seq
.........................................uagcaccauUugaaaucgguu..     3185      1          seq
.........................................uagcaccaucugaaaucgguua.     15495     0          seq
.........................................uagcaccauUugaaaucgguua.     919       1          seq
.........................................uagcaccaucugaaaucgguuau     1162      0          seq
.........................................uagcaccauUugaaaucgguuau     60        1          seq
..........................................agcaccaucugaaaucgg....     1         0          seq
..........................................agcaccauUugaaaucggu...     1         1          seq
..........................................agcaccaucugaaaucggu...     5         0          seq
..........................................agcaccaucugaaaucgguu..     47        0          seq
..........................................agcaccaucugaaaucgguua.     14        0          seq
..........................................agcaccauUugaaaucgguua.     2         1          seq
..........................................agcaccaucugaaaucgguuau     1         0          seq
...........................................gcaccaucugaaaucgguu..     7         0          seq
...........................................gcaccaucugaaaucgguua.     2         0          seq
```
